# Effects of Oil Pulling and Chlorhexidine Mouth Rinse on the Force Decay of Orthodontic Elastomeric Chains: A Comparative In Vitro Study

**DOI:** 10.7759/cureus.53456

**Published:** 2024-02-02

**Authors:** Keerthana Sivaraman, Rajasekaran UB, Neetika Prabu, Arun Deepak, Nagaland T, Anusha Sreedharan

**Affiliations:** 1 Orthodontics and Dentofacial Orthopaedics, RVS Dental College and Hospital, Coimbatore, IND; 2 Orthodontics, RVS Dental College and Hospital, Coimbatore, IND; 3 Public Health Dentistry, Chettinad Dental College and Research Institute, Chennai, IND

**Keywords:** mouth rinse, force decay, elastomeric chains, oil pulling, chlorhexidine

## Abstract

Background and objectives: Oil pulling is a traditional Indian folk remedy for maintaining oral hygiene among orthodontic patients. This study aimed to assess the effects of oil pulling and compare them with those of chlorhexidine (CHX) and distilled water on the force decay of elastomeric chains.

Methods: Twenty-one samples were tested in three groups. Each of these groups contained seven samples per group. The samples evaluated contained distilled water, 0.2% CHX, and sesame oil. The samples were dipped in various mouth rinses, and force degradation was measured using a dynamometer (dynamic universal testing machine, Instron 8801, Instron, Norwood, MA) during days 0, 1, 7, 14, 21, and 28. The significance level was considered at 1%.

Results: Force degradation was observed more in distilled water, followed by sesame oil, and less in CHX at the end of 28 days. Significant differences in values (p < 0.01) were found among the three groups in all the timelines in the study except on days 14 and 21.

Conclusion: Chlorhexidine showed the least amount of force decay, followed by oil pulling and distilled water. However, if oil pulling is practiced daily as a household remedy along with regular oral hygiene practices, it can save time and money and enhance general health.

## Introduction

Individuals with orthodontic appliances must exercise extra care when maintaining oral hygiene because appliances increase the buildup of bacteria around brackets and bands [1, 2]. Orthodontists must make extra efforts to provide preventive education to each patient. For those who are unable to maintain good oral hygiene, chemical plaque management should be employed in addition to mechanical control [3]. Chlorhexidine (CHX) is one of the most potent and extensively researched antibacterial mouth rinses. Despite being considered the ‘gold standard’, it has several adverse effects associated with its prolonged use, such as impaired taste perception and tooth discoloration. It also affects the physical and mechanical characteristics of certain orthodontic components, such as the staining of modules and the force deterioration of elastics over time [4-6].

Commercial mouthwashes can facilitate antibiotic resistance. This notion has motivated the search for natural products to preserve dental health. Dr. Karach F. popularized the practice of oil pulling with edible oils in contemporary medicine [7]. Oil pulling is the traditional Ayurvedic practice of gargling with oil to prevent decay, foul breath, bleeding gums, and cracked lips, in addition to strengthening teeth, gums, and jaws. It is known to have therapeutic local as well as systemic effects and is considered an effective alternative to CHX for routine oral hygiene practices [6, 8]. The procedure of oil pulling involves swishing a measured volume of oil around the mouth for a period, forcing the oil in between all the teeth and around the mouth. Examples of organic oils that are used include sunflower oil, sesame oil, and coconut oil [9]. Sesame oil has the following advantages over CHX: no staining, no lingering aftertaste, and no allergy. Sesame oil is readily available in most homes and is five to six times more affordable than CHX [10].

The British Society of Periodontology states that "antiplaque agents like CHX are useful for managing acute periods when cleaning is difficult but not needed as a routine" [11]. Additionally, it must be noted that the use of CHX mouthwash is licensed only for 30 days of use [12]. Therefore, for patients receiving fixed orthodontic therapy, a safe, cost-effective, and frequently used substitute for CHX mouth rinse is needed. Though oil pulling is an obsolete procedure, it should be reinstated as a regular dental hygiene measure.

Elastomeric chains have been widely used in orthodontics since the 1960s because they do not require patient cooperation and are relatively hygienic, affordable, and simple to use [13, 14]. Elastic devices are important sources for the transmission of force to teeth but are not considered ideal because the force diminishes with activation time, oral media, and other dietary-related characteristics [15].

However, there is no scientific research in support of oil pulling that compares the effects of force degradation on elastomeric chains. Hence, the present study was designed to assess the effects of oil pulling and compare them with those of CHX and distilled water for one month.

## Materials and methods

**Figure 1:**
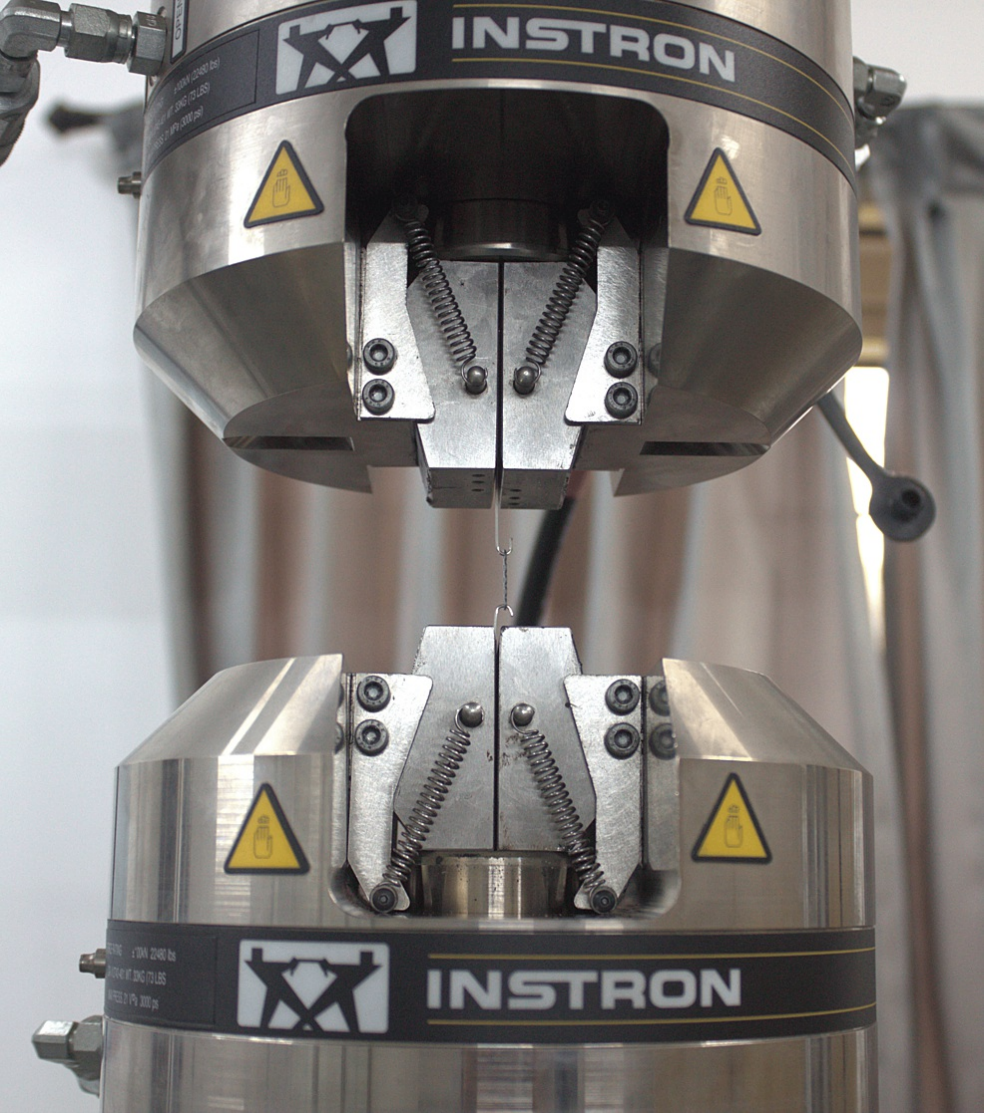
Dynamometer (dynamic universal testing machine, Instron 8801, Instron, Norwood, MA)

A laboratory study was conducted at the PSG Centre for Research and Consultancy, Coimbatore, India, for 28 days to test the force degradation of elastomeric chains using a dynamometer (dynamic universal testing machine, Instron 8801, Instron, Norwood, MA) (Figure 1).

The following armamentarium was used: distilled water, 0.2% CHX mouth rinse (Hexidrin brand), sesame oil (Idhayam brand), artificial saliva (Xerostat brand), and orthodontic elastomeric chains (short, American Orthodontics brand). This study did not involve the use of any animals, human data, or tissues; therefore, consent and ethical approval were not required.

**Figure 2:**
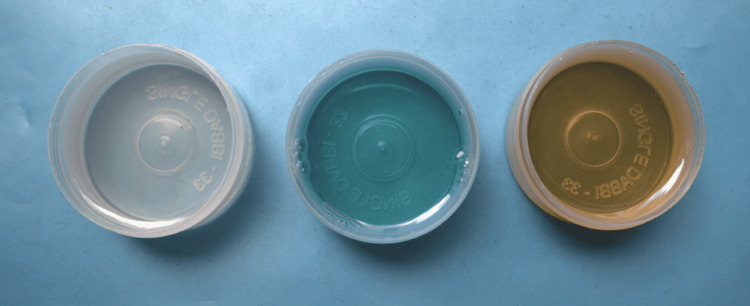
Control (distilled water) and experimental groups (chlorhexidine and sesame oil)

Twenty-one samples were tested in three groups. There were seven samples per group. The samples evaluated were distilled water (Group 1: control), 0.2% CHX (Group 2: experimental group), and sesame oil (Group 3: experimental group) (Figure 2).

**Figure 3:**
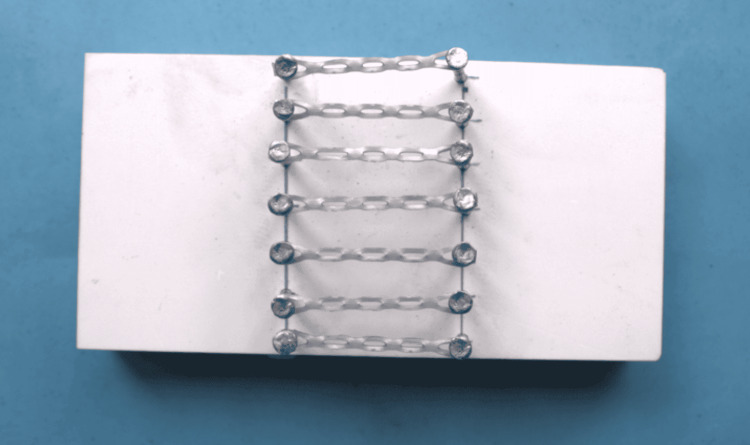
Orthodontic elastomeric chains in a fabricated template

A customized wooden template was fabricated with seven pairs of supporting rods arranged row-wise, with 23.5 mm gap between each set of rods. Short elastomeric chains of five links each were pre-stretched 1.5 times the original length and fixed to the supporting rod (Figure 3).

**Figure 4:**
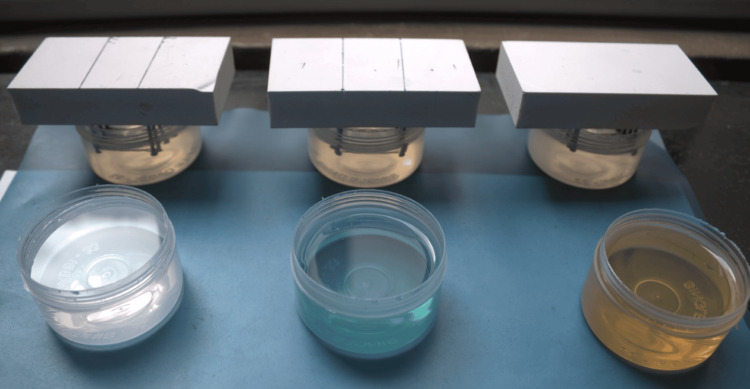
Samples immersed in artificial saliva

These were immersed in the artificial saliva solution at a controlled temperature (Figure 4).

The wooden template was dipped in the corresponding experimental and control solutions for one minute daily. These were then dipped in separate water baths for 10 seconds and placed back in the artificial saliva. The level of saliva in the template was verified every day to ensure the elastics were covered by this solution at all times. Six force measurements were taken during the experimental period at the following time intervals: days 0, 1, 7, 14, 21, and 28.

Statistical analysis

Data were entered in a Microsoft Excel sheet (Microsoft Corp., Redmond, WA), and statistical analysis was conducted using IBM SPSS software version 20.0 (IBM Corp., Armonk, NY). Mean and standard deviation were used to summarize the data. The effects of the groups (distilled water, CHX 0.2%, and sesame oil) and time (days 0, 1, 7, 14, 21, and 28) on force decay were analyzed using ANOVA and Tukey’s post hoc test. The significance level was considered at 1% (< 0.01).

## Results

Factors influencing the force decay of elastomeric chains were time duration, type of elastomeric chain, and exposure to test solutions. The maximum force (0.572 kg) was observed in CHX, followed by sesame oil (0.460 kg), and the least force (0.435 kg) was observed in distilled water at the end of 28 days (Table 1).

**Table 1:** Force (Kg) measured for elastomeric chains with different mouth rinses at different time periods

Time interval	Distilled water mean (SD)	Chlorhexidine mean (SD)	Sesame oil mean (SD)
Day 0	0.728 (0.10)	0.827 (0.02)	0.715 (0.03)
Day 1	0.524 (0.07)	0.647 (0.00)	0.533 (0.00)
Day 7	0.490 (0.05)	0.610 (0.01)	0.518 (0.05)
Day 14	0.471 (0.12)	0.587 (0.05)	0.487 (0.09)
Day 21	0.455 (0.05)	0.586 (0.05)	0.482 (0.00)
Day 28	0.435 (0.05)	0.572 (0.06)	0.460 (0.01)

When the groups were compared with one another in the same period, no statistical difference was found on days 14 and 21 (p > 0.01). Statistically significant differences in force levels were noted during the initial hours, during 24 hours, on day seven, and after day 28 (p < 0.01) (Table 2).

**Table 2:** One-way ANOVA test among the groups at different time intervals *Distilled water (Group 1: control), chlorhexidine (Group 2: experimental group), and sesame oil (Group 3: experimental group). # p < 0.01: significant

Timeline	Groups^*^	Mean	SD	95% confidence interval for mean		p-value^#^
				Lower bound	Upper bound	
Initial	1	.70100	.021517	.64755	.75445	0.001
	2	.80500	.018028	.76022	.84978	
	3	.70433	.007506	.68569	.72298	
24 hours	1	.51567	.022279	.46032	.57101	0.002
	2	.63100	.027074	.56374	.69826	
	3	.51367	.023671	.45486	.57247	
7 days	1	.43500	.054083	.30065	.56935	0.012
	2	.59333	.037859	.49929	.68738	
	3	.53767	.036828	.44618	.62915	
14 days	1	.45167	.045369	.33896	.56437	0.065
	2	.56000	.052000	.43082	.68918	
	3	.45867	.051598	.33049	.58684	
21 days	1	.43500	.030414	.35945	.51055	0.028
	2	.57300	.066461	.40790	.73810	
	3	.45233	.045347	.33969	.56498	
28 days	1	.42167	.019088	.37425	.46908	0.001
	2	.57267	.006429	.55670	.58864	
	3	.46000	.010000	.43516	.48484	

**Figure 5:**
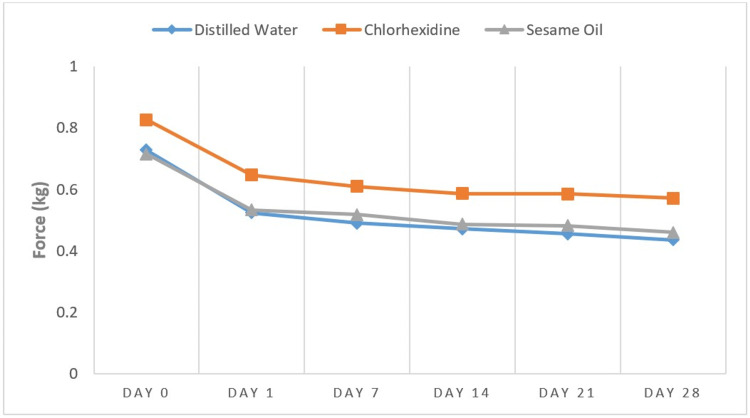
Force levels of various mouth rinses at each time interval

A sudden decline in the force levels during the initial 24 hours, followed by a gradual reduction of force over a period, was noted (Figure 5).

Multiple group comparison was done using Tukey’s post hoc test. Statistically significant differences (p < 0.01) were noted in force levels when comparing Group 1 with Group 2, Group 2 with Groups 1 and 3, and Group 3 with Group 2 during the initial hours; when comparing Group 1 with Group 2, Group 2 with Groups 1 and 3, and Group 3 with Group 2 during 24 hours; when comparing Group 1 with Group 2 and Group 2 with Group 1 on the seventh day; and Group 1 with Group 2, Group 2 with Groups 1 and 3, and Group 3 with Group 2 on the 28th day. No significant differences (p > 0.01) were noted in force levels when multiple groups were compared on days 14 and 21; when Group 1 was compared with Group 3 and Group 3 was compared with Group 1 during the initial hours, during 24 hours, and on the day 28; and when Group 1 was compared with Group 3, Group 2 with Group 3, and Group 3 with Groups 1 and 2 on the day seven (Table 3).

**Table 3:** Tukey’s post hoc test; multiple group comparisons *p < 0.01: significant

Dependent variable	(I) Group	(J) Group	Mean difference (I-J)	p-value^*^	
					Initial	1	2	-.104000^*^	.001	
3	-.003333	1.000				
2	1	.104000^*^	.001		
	3	.100667^*^	.001		
3	1	.003333	1.000		
	2	-.100667^*^	.001		
24 hours	1	2	-.115333^*^	.004	
		3	.002000	1.000	
	2	1	.115333^*^	.004	
		3	.117333^*^	.003	
	3	1	-.002000	1.000	
		2	-.117333^*^	.003	
7 days	1	2	-.158333^*^	.013	
		3	-.102667	.084	
	2	1	.158333^*^	.013	
		3	.055667	.508	
	3	1	.102667	.084	
		2	-.055667	.508	
14 days	1	2	-.108333	.112	
		3	-.007000	1.000	
	2	1	.108333	.112	
		3	.101333	.141	
	3	1	.007000	1.000	
		2	-.101333	.141	
21 days	1	2	-.138000^*^	.043	
		3	-.017333	1.000	
	2	1	.138000^*^	.043	
		3	.120667	.074	
	3	1	.017333	1.000	
		2	-.120667	.074	
28 days	1	2	-.151000^*^	.000	
		3	-.038333^*^	.033	
	2	1	.151000^*^	.000	
		3	.112667^*^	.000	
	3	1	.038333^*^	.033	
		2	-.112667^*^	.000	

## Discussion

Orthodontic appliances attached to tooth surfaces make it difficult to practice oral hygiene and act as additional bacterial plaque reservoirs. The enamel is demineralized as a result, leading to white spots, dental cavities, and gingivitis [1]. To prevent enamel demineralization, various mouth rinses are being prescribed. Out of these, chlorhexidine is a highly effective synthetic antibacterial agent [3]. Oil pulling can be used as an alternative method to maintain oral hygiene in orthodontic patients [9].

In the present study, elastomeric chain usage was considered to be 28 days because this matched the average amount of time between orthodontic consultations, as Motta et al. [16] and Pithon et al. [17] noted. The elastomeric chain segment was kept submerged in artificial saliva because force degradation in a humid medium is substantially greater than in a dry environment [18-20]. Short elastomeric chains were used to maintain a higher percentage of force over time [21].

The latex elastics in the present investigation displayed a force relaxation in the range of 22%-27% in the first 24 hours. Force levels at the end of 24 hours were 0.64 kg, 0.53 kg, and 0.52 kg in chlorhexidine (Group 2), sesame oil (Group 3), and distilled water (Group 1), respectively (Table 1). This result was similar to those of Pithon et al. [17], with a 20%-30% force decay. In contrast, Singh et al. [21] and Sam et al. [22] showed that the rates of force decay were 50% to 70% and 17% to 24%, respectively, for the first 24 hours. There was a large decrease in force during the first 24 hours, followed by mostly stable levels of force up to four weeks (Figure 5). This result is in line with those of Pithon et al. [17], Sufarnap et al. [23], Issa et al. [24], Samuels et al. [25], and Balhoff et al. [26].

However, results may vary depending on whether other biological components are present in the oral environment. Because this study was conducted in vitro under static conditions, the elastomeric chain’s performance could not replicate the degradation observed in vivo. Further studies in clinical settings, where the oral environment is varied because of dietary habits, microbial activity, different stretching conditions, and different brands of elastomeric chains, are necessary. This would help us better comprehend elastic materials’ physical characteristics in various clinical settings. Studies using different organic oils and herbal mouth rinses should be carried out in the future so that they can be used as an alternative to chlorhexidine in assessing the force decay of orthodontic elastomeric chains.

## Conclusions

Chlorhexidine showed the least amount of force decay compared to oil and distilled water. Similarly, oil pulling had a significantly lower force decay on elastomeric chains compared to distilled water. It is thus concluded that, though CHX showed the least amount of force decay, it had certain adverse effects on prolonged usage. Hence, oil pulling can be recommended as a preventive oral hygiene practice with minimal adverse effects in fixed orthodontic patients because the treatment takes place over a longer duration. If oil pulling is practiced daily as a household remedy along with regular oral hygiene practices, it can save time and money and enhance general health.
